# Authors’ reply

**Published:** 2010

**Authors:** Varsha Nandedkar, Anil R Joshi, Namrata Kabra, Neha N Deshpande

**Affiliations:** Department of Ophthalmology, Government Medical College, Aurangabad - 431 001, India; 1 Department of Pathology, Government Medical College, Aurangabad - 431 001, India

Dear Editor,

First, we appreciate the details with which Chalisgaonkar *et al*.[1] have read our case report[2] and searched the literature of retinoblasotoma in adults.

In the case report by Biswas *et al*.,[3] on the computed tomogram (CT), ’a speck of calcification’ was noted. No gross calcified areas were found. In the remaining cases no calcification was noted.

In all case reports of retinoblastoma in adults, retinocytoma was diagnosed as benign appearing areas in malignant tumor tissue and not as pre-existing lesions that turned malignant on follow-up.[4] In our case no such benign appearing cells were noted in the malignant tissue, in the multiple tissue sections that were taken.[1]

Ours is the only case reported in literature where retinoblastoma was found in pregnancy. There is one case reported by Takahashi *et al*.,[5] six months postpartum. We have stated that new cases and further study is needed for establishing this relationship. Pregnancy *may* have an effect on retinoblastoma due to circulating mutagens and immunosuppression.[6]
